# Behavioral abnormalities with disruption of brain structure in mice overexpressing VGF

**DOI:** 10.1038/s41598-017-04132-7

**Published:** 2017-07-05

**Authors:** Takahiro Mizoguchi, Hiroko Minakuchi, Mitsue Ishisaka, Kazuhiro Tsuruma, Masamitsu Shimazawa, Hideaki Hara

**Affiliations:** Molecular Pharmacology, Department of Biofunctional Evaluation, Gifu Pharmaceutical University, Gifu, Japan

## Abstract

VGF nerve growth factor inducible (VGF) is a neuropeptide induced by nerve growth factor and brain-derived neurotrophic factor. This peptide is involved in synaptic plasticity, neurogenesis, and neurite growth in the brain. Patients with depression and bipolar disorder have lower-than-normal levels of VGF, whereas patients with schizophrenia and other cohorts of patients with depression have higher-than-normal levels. VGF knockout mice display behavioral abnormalities such as higher depressive behavior and memory dysfunction. However, it is unclear whether upregulation of VGF affects brain function. In the present study, we generated mice that overexpress VGF and investigated several behavioral phenotypes and the brain structure. These adult VGF-overexpressing mice showed (a) hyperactivity, working memory impairment, a higher depressive state, and lower sociality compared with wild-type mice; (b) lower brain weight without a change in body weight; (c) increased lateral ventricle volume compared with wild-type mice; and (d) striatal morphological defects. These results suggest that VGF may modulate a variety of behaviors and brain development. This transgenic mouse line may provide a useful model for research on mental illnesses.

## Introduction

Mental illnesses are high prevalence^1^. Suicide is a common cause of death worldwide and a leading cause of death in young people^2^. Suicide rates are much higher in people with mental health problems^3^. Although many studies have investigated candidate genes for mental illnesses, these disorders still have no cure^4–8^. Animal models that reflect side and symptom of mental illness in humans are needed to allow new approaches to the study of these diseases^9^.

VGF nerve growth factor inducible (VGF) is a peptide precursor that is processed into biologically active peptides such as TLQP-21 and AQEE-30^10, 11^. This neuropeptide was first identified as a nerve growth factor (NGF)-induced protein in PC12 cells (a cell line derived from a pheochormocytoma of rat adrenal medulla)^12^. Later studies demonstrated that VGF is also upregulated by other neurotrophic factors, including brain-derived neurotrophic factor (BDNF), epidermal growth factor (EGF), and neurotrophin-3 (NT-3) in PC12 cells and in primary cultures of cortical and hippocampal neurons^13, 14^. The effects of VGF are associated with the processing of pro-BDNF to form mature BDNF, and the phosphorylation of tyrosine protein kinase B (TrkB)^15, 16^. Hence, it has been suggested that the effects of VGF are closely implicated with BDNF signaling, such as the autoregulatory BDNF loop^15, 16^.

VGF may play roles in the regulation of synaptic plasticity, neurogenesis, and neurite growth in the brain^17–19^. Patients with depression and bipolar disorder express lower levels of VGF, while patients with schizophrenia and other cohorts of patients with depression express higher levels of VGF^20–22^. Based on these clinical findings, the phenotype of VGF germline knockout mice was investigated. These mice exhibited an increase in depressive behaviors in the tail suspension test and forced swimming test and memory failure in the Morris water maze test and contextual fear conditioning without a change in locomotor activity^15, 22, 23^. These mice also show abnormalities in sensitivity to lithium and long-term potentiation (LTP)^15, 22^. Additionally, the forebrain VGF knockout mice exhibited fear memory impairment^16^.

Based on these results, the downregulation of VGF has been postulated to contribute to the pathogenesis of mental illness^15, 22, 23^. However, the mechanism whereby VGF upregulation contributes to mental illness is unknown. To address this question, we developed a line of VGF-overexpressing mice and investigated the roles of VGF in behavior, brain formation, and neurotrophin regulation.

## Materials and Methods

### Animals

**Figure 1:**
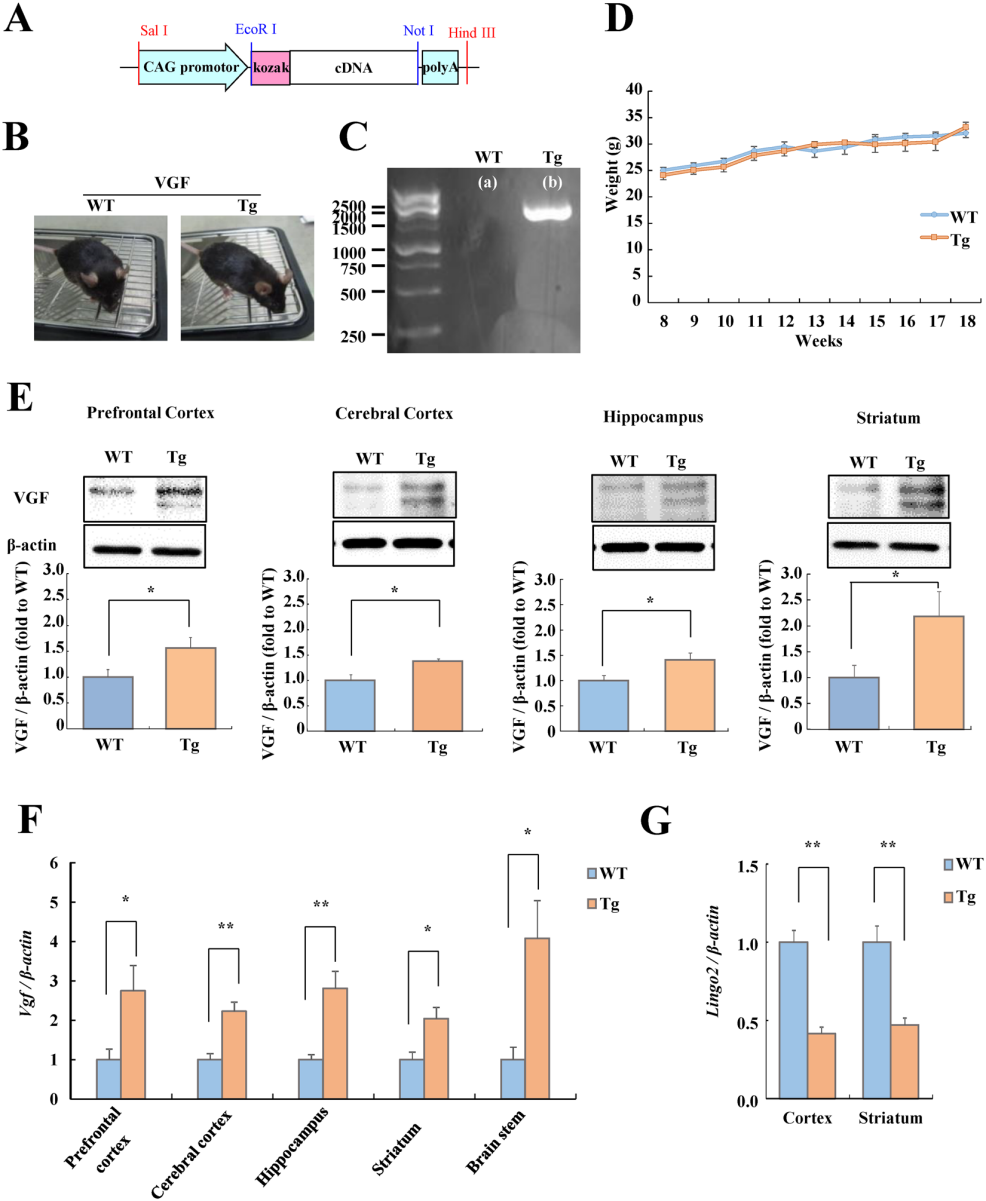
Generation of VGF-overexpressing mice. (**A**) Schematic representation of the VGF transgene used to generate VGF-overexpressing mice. (**B**) Photographs of WT (left) and VGF-overexpressing (right) mice. (**C**) The VGF transgene is detectable by PCR in only the VGF-overexpressing mice. (**D**) Comparison of the body weights of WT and VGF-overexpressing mice at 8 to 18weeks of age. Data are expressed as the mean ± SEM (WT, n = 6; Tg, n = 4). (**E**) Expression levels of VGF in several brain regions in WT and VGF-overexpressing mice were evaluated by western blotting relative to β-actin levels. Data are expressed as the mean fold difference versus WT mice ± SEM (WT, n = 6; Tg, n = 5). (pre: t = −2.281, df = 9, *p* = 0.048, ctx: t = −2.970, df = 9, *p* = 0.016, hip: t = −2.466, df = 9, *p* = 0.03 6, str: t = −2.312, df = 9, *p* = 0.046). Representative bands from the western blotting of VGF and β-actin are shown at the top. *p < 0.05 vs. WT mice (Student’s *t*-test). The cropped blots are used in this Figure and full-length blots are presented in Supplementary Figure S1. (**F**) Expression levels of *Vgf* mRNA in several brain regions in WT and VGF-overexpressing mice were evaluated by real-time RT-PCR relative to *β-actin* levels. Data are expressed as the mean fold difference versus WT mice ± SEM (WT, n = 4; Tg, n = 4). (pre: t = −2.512, df = 6, *p* = 0.046, ctx: t = −4.429, df = 6, *p* = 0.004, hip: t = −4.053, df = 6, *p* = 0.007, str: t = −3.005, df = 6, *p* = 0.024, stem: t = −3.048, df = 6, *p* = 0.023). *p < 0.05, **p < 0.01 vs. WT mice (Student’s *t*-test). (**G**) Expression levels of *lingo2* mRNA in several brain regions, retina, and optic nerve in WT and VGF-overexpressing mice were evaluated by real-time RT-PCR (ctx: t = 6.844, df = 6, *p* < 0.001, str: t = 4.740, df = 6, *p* = 0.003).

VGF-overexpressing mice (BDF1: a cross between female C57BL/6 and male DBA/2) were generated by pronuclear microinjection of BDF1 embryos followed by backcrossing of mice for more than 7 generations onto BDF1 background. A sequence of 1854 nucleotides from the CAG promoter was amplified by polymerase chain reaction ﻿(PCR) with pCAGGS vector and cloned using the Hind III-SalI sites. In addition to the CAG promoter (CMV-enhancer/chicken beta-actin promoter) and cDNA sequence for VGF, this transgene fragment contained a polyadenylation site for mRNA stabilization (Fig. 1A). Wild-type (WT) and VGF-overexpressing mice were generated by heterozygous VGF-overexpressing mice and BDF1 mice (SLC, Shizuoka, Japan). We used WT littermates as a control group of VGF-overexpressing mice. The animals were housed in same sex littermate groups. The genotype of each mouse was confirmed by PCR. The target gene (VGF transgene) was amplified by 35 cycles of PCR using the following primers: VGF forward primer, 5′-CCTACAGCTCCTGGG-3′; VGF reverse primer, 5′-AGAGGGAAAAAGATCRCAGTGGTAT-3′. Six transgenic founders were produced (lines 403, 411, 413, 423, 429, and 434). Three of these lines (lines 403, 413, and 434) exhibited impaired breeding. Two lines (lines 411 and 423) did not display increased expression of VGF, as determined by real-time RT-PCR (data not shown). After these preliminary experiments, line 429 was selected for further study. The male animals (8 to 18 weeks old) were housed at 24 °C under a 12 h light-dark cycle (lights on from 8:00 to 20:00); all had ad libitum access to food (CE-2; CLEA Japan inc, Tokyo, Japan) and water. All procedures relating to animal care were approved and monitored by the Institutional Animal Care and Use Committee of Gifu Pharmaceutical University. All efforts were made to minimize suffering and the number of animals used. All procedures relating to animal care conformed to animal care guidelines issued by the National Institutes of Health.

### Western blot analysis

Each naïve mouse (18 weeks old) was decapitated, and its brain was quickly removed and placed on a cooled plate, where the prefrontal cortex, cerebral cortex, hippocampus, and striatum were rapidly dissected. The same mice were used across brain regions. The tissues were homogenized in ice-cold tissue lysis buffer (50 mM Tris-HCl [pH 8.0] containing 150 mM NaCl, 50 mM EDTA, 1% Triton X-100) and protease/phosphatase inhibitor mixture (Sigma-Aldrich, St. Louis, MO, USA) using a homogenizer (Physcotron; Microtec Co. Ltd., Funabashi, Japan). The lysate was centrifuged at 10 000 rpm for 20 min, and the supernatant collected for use in the experiments. The protein concentration was determined by comparison with known concentrations of bovine serum albumin using a BCA Protein Assay kit (Thermo Fisher Scientific, Waltham, MA, USA). Lysates were solubilized in sodium dodecyl sulfate sample buffer, separated on a 5–20% sodium dodecyl sulfate-polyacrylamide gradient gel (Wako Pure Chemical Industries, Osaka, Japan), and transferred to polyvinylidene difluoride membrane (Immobilon-P; Milipore Corp., Billerica, MA, USA). The membranes were blocked for 30 min or 1 h at room temperature with 5% skim milk or Blocking One P (Nakarai Tesque, Inc., Kyoto, Japan). After blocking, the membranes were washed in 10 mM Tris-buffered saline with 0.05% Tween20 and then incubated overnight at 4 °C with the primary antibody. The membranes were washed in 10 mM Tris-buffered saline with 0.05% Tween20 and incubated for 1 h at room temperature in horseradish peroxidase rabbit anti-goat (Thermo Fisher Scientific) diluted 1:75 000, rabbit anti-mouse (Thermo Fisher Scientific) diluted 1:2000, or goat anti-rabbit (Thermo Fisher Scientific) diluted 1:2000. Immunoreactive bands were developed using ImmunoSter LD (Wako) and visualized with the aid of a digital imaging system (LAS-4000UVmini; Fujifilm, Tokyo, Japan). The primary antibodies used are as follows: goat polyclonal anti-VGF (sc-10383; Santa Cruz Biotechnologies, CA, USA) diluted 1:400, mouse monoclonal anti-β-actin (Sigma-Aldrich) diluted 1:5000, and rabbit monoclonal anti- poly ADP-ribose polymerase (PARP) diluted 1:1000 (Cell signaling, Danvers, MA, USA).

### Real-time RT-PCR analysis

VGF overexpression in the brain was confirmed by real-time RT-PCR analysis. Samples were collected from the prefrontal cortex, cerebral cortex, hippocampus, striatum, and brain stem of VGF-overexpressing mice and WT mice (18 weeks old) using the method described for Western blot analysis. Total RNA was isolated according to the manufacturer’s protocol for NeuroSpin RNA II (Takara BIO INC., Shiga, Japan). First-strand cDNA was synthesized from total RNA in a 20-µL reaction mixture using the PrimeSprict RT reagent Kit (Takara). Real-time RT-PCR was performed with a Thermal Cycler Dice Real Time System II (Takara) using SYBR Premix Ex Taq II (Takara). The PCR protocol consisted of a 30-sec denaturation step at 95 °C, followed by a two-step PCR comprising 5 sec at 95 °C and 30 sec at 60 °C, with 40 cycles for VGF and β-actin. For VGF, the forward primer was 5′-CAGGCTCGAATCCGAAAG-3′ and the reverse primer was 5′-CTTGGATAAGGGTGTCAAAGTCTCA-3′. For β-actin, the forward primer was 5′-CATCCGTAAAGACCTCTATGCC-3′, and the reverse primer was 5′-ATGGAGCCACCGATCCACA-3′. Quantitative real-time RT-PCR analysis was performed using a Thermal Cycler Dice Real Time System TP 800 (Takara).

### Histological analysis

**Figure 2:**
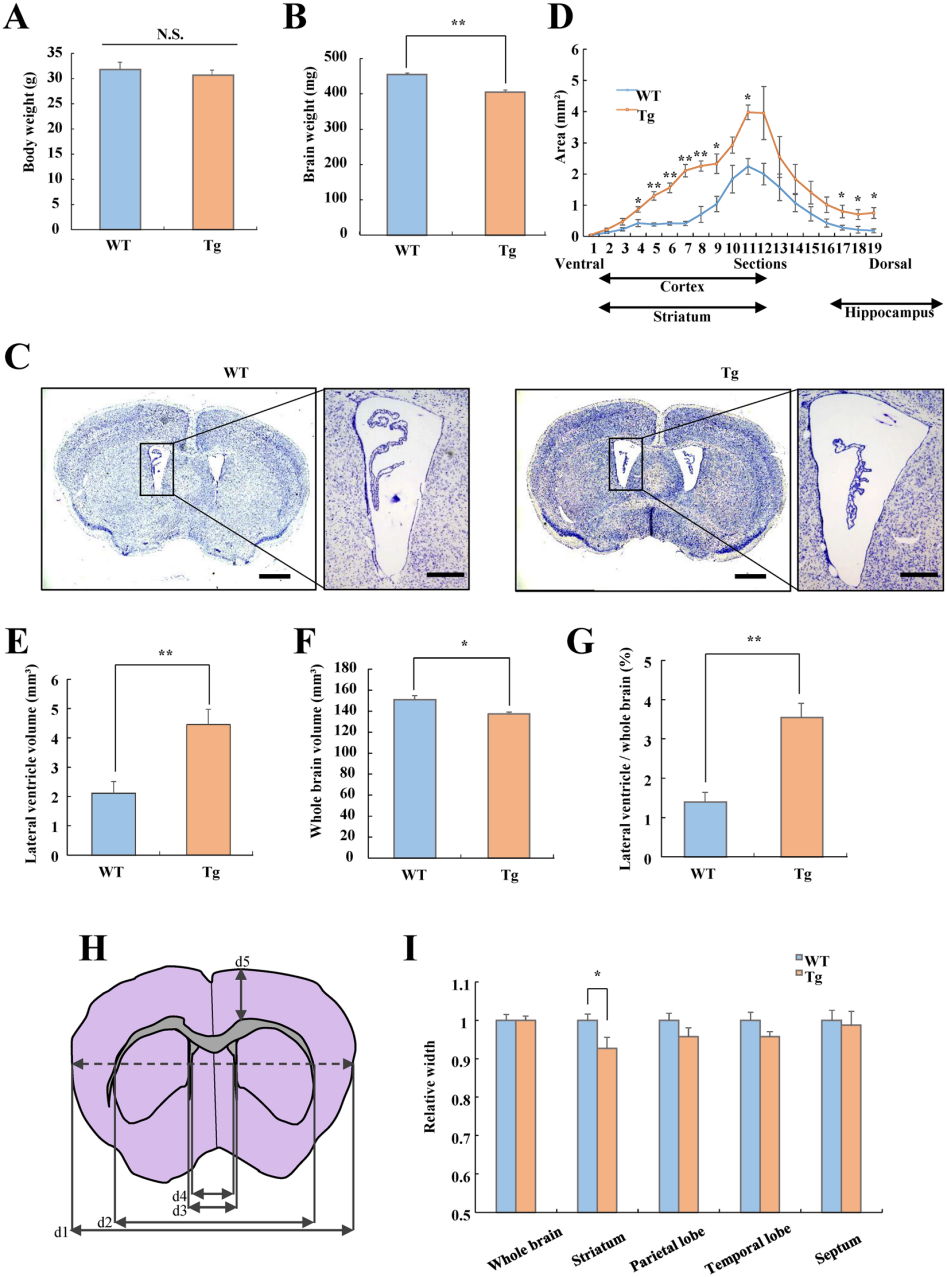
Histological analysis of WT and VGF-overexpressing mice. Body weight (t = 0.643, df = 21, *p* = 0.527) (**A**) and brain weight (**B**) of adult WT and VGF-overexpressing mice (t = 6.136, df = 21, *p* < 0.001). Data are expressed as the mean ± SEM (WT, n = 10; Tg, n = 13), **p < 0.01 vs. WT mice (Student’s *t*-test). (**C**) Representative photomicrographs show coronal sections stained with cresyl violet in WT and VGF-overexpressing mice. Scale bar = 1000 µm. Right panels show enlargements of the lateral ventricle region. Scale bar = 300 µm. (**D**) The lateral ventricle region in WT and VGF-overexpressing mice is visualized per section in a ventral to dorsal manner. Genotype; F (1, 8) = 13.491, *p* < 0.001, section; F (18, 144) = 32.716, *p* < 0.001, genotype × section; F (18, 144) = 3.223, *p* < 0.001, two-way ANOVA with repeated measures (4: t = −3.015, df = 8, *p* = 0.017, 5: t = −6.312, df = 8, *p* < 0.001, 6: t = −6.789, df = 8, *p* < 0.001, 7: t = −8.040, df = 8, *p* < 0.001, 8: t = −5.145, df = 8, *p* = 0.001, 9: t = −3.260, df = 8, *p* = 0.012, 11: t = −5.042, df = 8, *p* = 0.001, 17: t = −2.375, df = 8, *p* = 0.045, 18: t = −2.579, df = 8, *p* = 0.033, 19: t = −3.039, df = 8, *p* = 0.016). In the same mice as above, the lateral ventricle volume (t = −3.560, df = 8, *p* = 0.007) (**E**), whole brain volume (t = 3.177, df = 8, *p* = 0.013) (**F**), and ratio of lateral ventricle volume to whole brain volume (t = −4.156, df = 8, *p* = 0.003) (**G**) was measured. Data are expressed as the mean ± SEM (WT, n = 5; Tg n = 5), *p < 0.05, **p < 0.01 vs. WT mice (Student’s *t*-test). (**H**) Diagram of the brain section indicates the five regions measured for length using cresyl violet. The width of each brain region was defined as follows: whole brain = d4, striatum = d3 − d2, motor cortex = d5, somatosensory cortex = d4–d3, and septum = d4. (**I**) Width of the whole brain, striatum, parietal lobe, temporal lobe, and septum of WT and VGF-overexpressing mice (whole brain: t = −0.020, df = 18, *p* = 0.984, striatum: t = 2.315, df = 18, *p* = 0.033, temporal lobe: t = 1.613, df = 18, *p* = 0.124, parietal lobe: t = 1.466, df = 18, *p* = 0.160, septum: t = 0.292, df = 18, *p* = 0.774). Data are expressed as the mean ± SEM (WT, n = 11; Tg, n = 9), *p < 0.05 vs. WT mice (Student’s *t*-test).

Each mouse (12 to 18 weeks old) was anesthetized with sodium pentobarbital (50 mg/kg) and perfused with saline until the outflow became clear. The perfusate was then changed to 0.1 M phosphate buffer (PB; pH 7.4) containing 4% paraformaldehyde (Wako) for 8 min. The brain was quickly removed and kept immersed for at least 24 h at 4 °C in the same fixative. The fixed specimens were dehydrated through a graded series of ethanol and xylene and finally embedded in paraffin. For Nissl staining, paraffin-embedded specimens were cut at 5-µm thickness; the sections were mounted on microslide glass (Matsunami Glass Ind. Ltd., Osaka, Japan) and deparaffinized. Next, they were stained in cresyl violet for 10 to 20 min and dehydrated using absolute ethanol and xylene. Images were taken under a light microscope (Keyence, Osaka, Japan). For determination of the number of neurons in the striatum and hippocampus, at least two coronal sections per animal were scanned using a 40x objective. To determine the volumes of the lateral ventricle, striatum, and hippocampus, we applied Cavalieri’s principle (volume = s_1_d_1_ + s_2_d_2_ + … s_n_d_n_, s = area and d = distance between two sections) with an interval of 140 µm between the sections^24, 25^. Nissl-stained coronal sections were prepared and outlined using the 4x objective at the following positions relative to the bregma: lateral ventricle and whole brain, + 1.94 to −2.18 mm; striatum and cerebral cortex, +1.70 to −0.22 mm; hippocampus, −0.94 to −4.04 mm. For analysis of the width of several brain regions, the length of d1–d5 was measured at the bregma, + 0.50 to −0.10 mm (Fig. 2H). The width of each brain region was defined as follows: whole brain = d4, striatum = d3−d2, parietal lobe = d5, temporal lobe = d4–d3, and septum = d4. The mouse brain atlas^26^ was used to locate each brain region. The measurement was performed using ImageJ software (National Institutes of Health, Bethesda, MD, USA) in a blinded manner by a single observer (T.M.).

### Behavioral tests

All behavioral experiments were performed between 9:00 a.m. and 8:00 p.m. except for the 24 hr locomotor activity test using mice (8 to 18 weeks old). The same mice were used in open field test, Y-maze test, tail suspension test, forced swimming test, and passive avoidance test in this order. Moreover, other mice were used in locomotor activity test. We performed social interaction test with mice which we used for locomotor activity test and other mice. Locomotor activity test was performed before social interaction test. Each test was separated from each other by 1 day at least.

### Open field test

Each mouse was placed in the periphery of the open field apparatus (30 cm long × 30 cm wide × 15 cm high) at about 300 lux. The total distance moved in the arena and the time spent in the center (15 cm long × 15 cm wide) was recorded for 2 h using a computer-operated EthoVision XT system (Noldus, Wageningen, the Netherlands). The number of jumping behavior was also scored.

### Locomotor activity test

The locomotor activity test was conducted as previously described^27^. To measure locomotor activity in a familiar environment, a mouse was placed in a plastic home cage (24.5 cm long × 17.5 cm wide × 12.5 cm high) with sawdust bedding on the floor, food, and water. The term “home cage” is used to indicate a cage that is the same as that in which an animal is usually housed, i.e., the same size and same color. Animals were placed in the home cages at 12:00 and left there for 48 hr. Locomotion was measured every hour for 1 day after 24 h using a digital counter with an infrared sensor (NA-ASS01; Neuroscience, Inc., Tokyo, Japan).

### Y-maze test

The Y-maze test was performed as previously described with minor modification^28^. The apparatus consisted of three arms (10 cm long × 40 cm wide × 12 cm high) made of gray plastic. Each mouse was placed at the end of one fixed arm and allowed to explore freely for 8 min. The sequence of arm entries was recorded manually. An alternation was defined as consecutive entry into each of the 3 arms. The percentage of alternation was calculated as follows: [actual alternations/(total number of entries − 2)] × 100.

### Tail suspension test

The tail suspension test was conducted as previously described with minor modification^27^. The tail of each mouse was suspended using adhesive tape at an attitude of 50 cm for 7 min. The immobility time was measured automatically by the Ethovision XT system. Mice were judged to be immobile when the mobility score of the system was less than 10%.

### Forced swimming test

The forced swimming test was conducted as previously described with minor modification^27^. Each mouse was placed in a plastic cylinder (14-cm diameter) filled with 16 cm of water (24 ± 1 °C) for 7 min, and the immobility time was measured manually during the last 4 min. Mice were judged to be immobile when they remained floating passively in the water, making only small movements to keep their heads above the water. The measurement of immobility time was performed in a blind manner by a single observer (T.M.).

### Social interaction test

The social interaction test was conducted as previously described with minor modification^28^. Two mice of the same genotype that were bred in different cages were placed in a plastic cage (24.5 cm long × 17.5 cm wide × 12.5 cm high) and allowed to explore freely for 10 min. The total duration of the social interaction was measured using a video camera. Social interaction was defined as the following behaviors: sniffing, genital investigation, grooming, and wrestling.

### Passive avoidance test

The passive avoidance test was conducted as previously described^29^. The apparatus consisted of a light chamber (15.5 cm long × 9.6 cm wide × 18-cm height) and a dark chamber (32 cm long × 32 cm wide × 27 cm high). The two chambers were separated by a sliding plastic door. On the first day (habituation day), each mouse was placed in the light chamber and allowed to explore for 30 sec. The plastic door was then opened. When mice entered the dark chamber with all four paws, the plastic door was closed and the mice were allowed to explore the dark chamber for 30 sec. On the second day (training day: 24 h after the start of the first day), the trial was performed in the same manner as on day 1, except that mice received a foot shock (0.16 mA, 2 sec) when they entered the dark chamber with all four paws. On the third day (test day: 24 h after the start of the second day), the trial was performed as on day 1. In all trials, the latency to enter the dark chamber was measured until 600 sec.

### Statistical analysis

**Figure 3:**
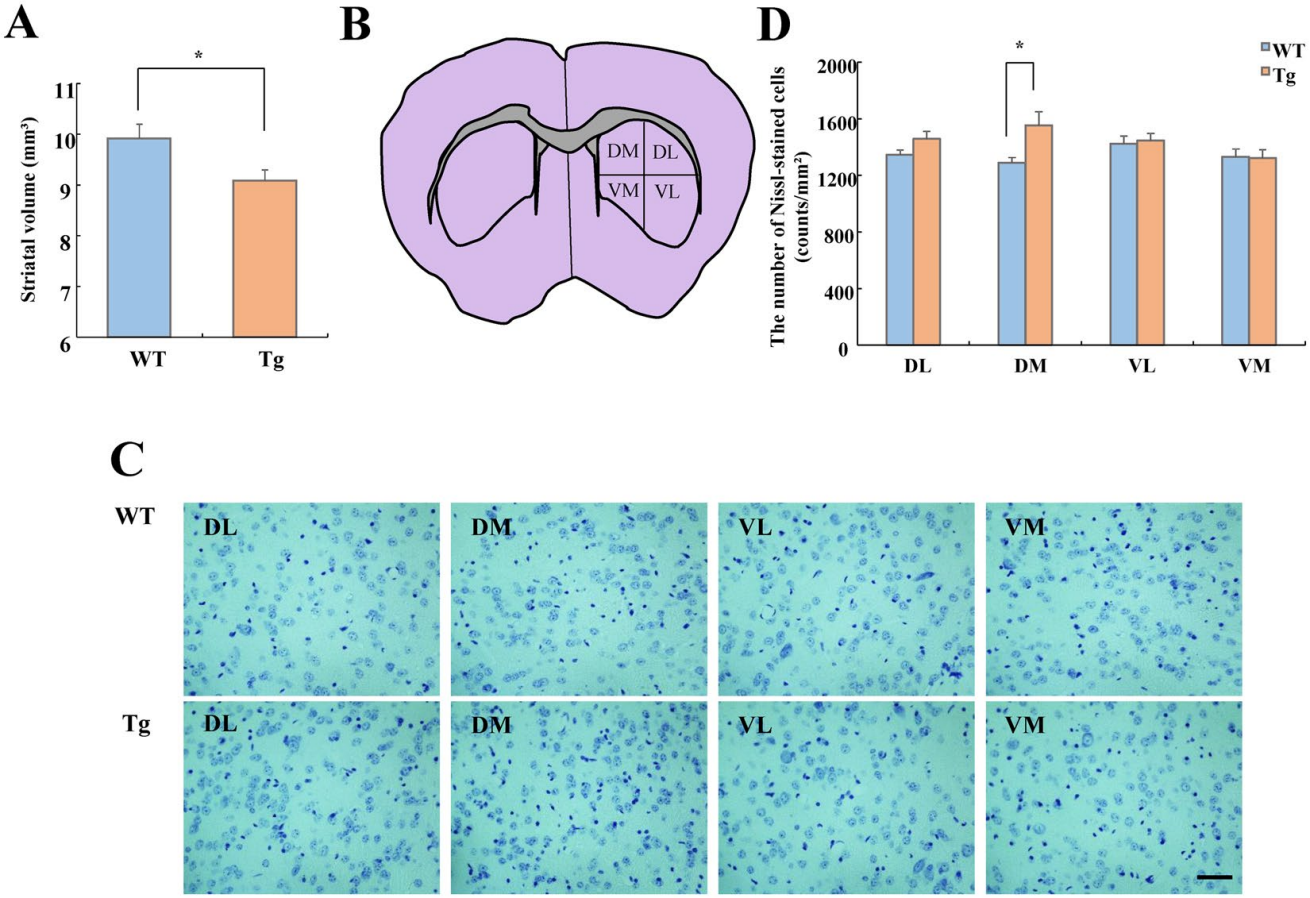
Histological analysis of the striatum in WT and VGF-overexpressing mice. (**A**) Striatal volume in WT and VGF-overexpressing mice (t = 2.412, df = 9, *p* = 0.039). Data are expressed as the mean ± SEM (WT, n = 5; Tg, n = 6), *p < 0.05 vs. WT mice (Student’s *t*-test). (**B**) Diagram of the striatum indicates the four regions in which cells were counted using cresyl violet. (**C**) Coronal sections of the striatum were stained with cresyl violet. Representative photomicrographs show coronal sections of four quadrants (DL, DM, VL, and VM) in WT (upper) and VGF-overexpressing (lower) mice. Scale bar = 50 µm. (**D**) The number of Nissl-stained cells was counted for each of the four quadrants (DL: t = −1.760, df = 18, *p* = 0.095, DM: t = −2.723, df = 18, *p* = 0.014, VL: t = −0.289, df = 18, *p* = 0.776, VM: t = 0.123, df = 18, *p* = 0.903). Data are expressed as the mean ± SEM (WT, n = 11; Tg, n = 9), *p < 0.05 vs. WT mice (Student’s *t*-test). DM, dorsal medial; DL, dorsal lateral; VM, ventral medial; VL, ventral lateral.

All values are expressed as the mean ± standard error. Quantitative variables were statistically analyzed using the Student two-tailed *t*-test or paired *t*-test for group comparisons and Mann–Whitney *U*-test for nonparametric values. Two-way ANOVA with repeated measures was used to analyze differences between groups (Figs 2D, 3A,F and H). *p*-values less than 0.05 were considered statistically significant. All statistical analyses were performed using Statistical Package for the Social Sciences 15.0 J for Windows (SPSS Japan, Inc., Tokyo, Japan).

## Results

### Generation of VGF-overexpressing mice

We generated VGF-overexpressing mice (Fig. 1B; right) and WT mice (Fig. 1B; left) by introducing a transgene into the anterior nucleus of the fertilized egg. There was no evident difference in the number of fetuses between VGF-overexpressing mice and WT mice (data not shown). The resulting VGF-overexpressing mice were identified by PCR analysis (Fig. 1C). There were no significant differences in body weight between adult VGF-overexpressing mice and WT mice aged 8 to 18 weeks (Fig. 1D). To examine the expression of VGF protein and mRNA in VGF-overexpressing mice and WT mice, we performed western blotting and real-time RT-PCR (Fig. 1E,F). The expression of VGF protein and mRNA in VGF-overexpressing mice was elevated in various brain regions compared with WT mice (Fig. 1E,F). We investigated only one line of the VGF-overexpressing mice, therefore cannot rule out the effect of transgene integration site on these results. Nextly, we identified only one integration site, which is located in the 2 nd intron of leucine rich repeat and Ig domain containing 2 (lingo 2) gene on mouse chromosome 4, and the expression level of *lingo 2* mRNA was decreased in VGF-overexpressing mice (Fig. 1G).

### Morphological changes in brains of VGF-overexpressing mice

The brain weight of adult VGF-overexpressing mice was significantly lower than that of age-matched WT mice, with no difference in body weight (Fig. 2A,B). Moreover, the brain weight to body weight ratio of VGF-overexpressing mice was less than WT mice (*p* = 0.052, data not shown). Brain-section analysis with cresyl violet staining revealed that the lateral ventricle was larger in VGF-overexpressing mice than in WT mice (Fig. 2C–E). This enlargement was most notable in the ventral region (Fig. 2D). The whole-brain volume was smaller and the ratio of the volume of the lateral ventricle to the whole brain was larger in VGF-overexpressing mice than in WT mice (Fig. 2F,G). Because the enlargement of lateral ventricle was most notable in the ventral region, we measured the size of brain regions located in the ventral region. The schematic diagram shown in Fig. 2H indicates the dimensions measured in the ventral region. The results indicate that the striatum of VGF-overexpressing mice was smaller than that of WT mice. However, there were no differences in the size of the cortex or septum (Fig. 2I).

We also investigated the volumes of the cortex, striatum, and hippocampus. VGF-overexpressing mice exhibited a reduced striatal volume without a change in the cortical or hippocampal volumes (Fig. 3A; Supplementary Fig. 3A,B). The reduced striatal volume led us to investigate the histology of the striatum in detail. It is reported that there are several striatal sectors mostly defined by their predominant cortical inputs^30^. We subdivided the striatum into four compartments to compare the dorsal-ventral and lateral-medial aspects of this structure as described previously (Fig. 3B)^31^. The number of Nissl-positive neuronal cells in coronal sections was compared between VGF-overexpressing mice and WT mice. Compared to WT mice, the density of neurons in VGF-overexpressing mice was significantly higher in the dorsal medial (DM) region but not in other regions (Fig. 3C,D). Additionally, the neuron density did not differ from that of WT in the hippocampus (Supplementary Fig. 3D). We also evaluated the expression level of cleaved PARP by Western blot analysis to determine whether apoptosis occurs in the striatum of VGF-overexpressing mice. Cleaved PARP was not detected in the striatum of WT or VGF-overexpressing mice (Supplementary Fig. 2). Apoptosis did occur in the positive control for apoptosis (NB-1RGB cells treated with UV-A [10 J/cm²] irradiation) (Supplementary Fig. 2)^32^.

### Changes in neurotrophin expression levels in brains of VGF-overexpressing mice

We evaluated the effect of VGF overexpression on the expression of NGF and BDNF in several brain regions. The expression levels of NGF and BDNF did not differ between VGF-overexpressing mice and WT mice (Supplementary Fig. 4A,B).

### VGF-overexpressing mice exhibit hyperactivity and less anxiety

**Figure 4:**
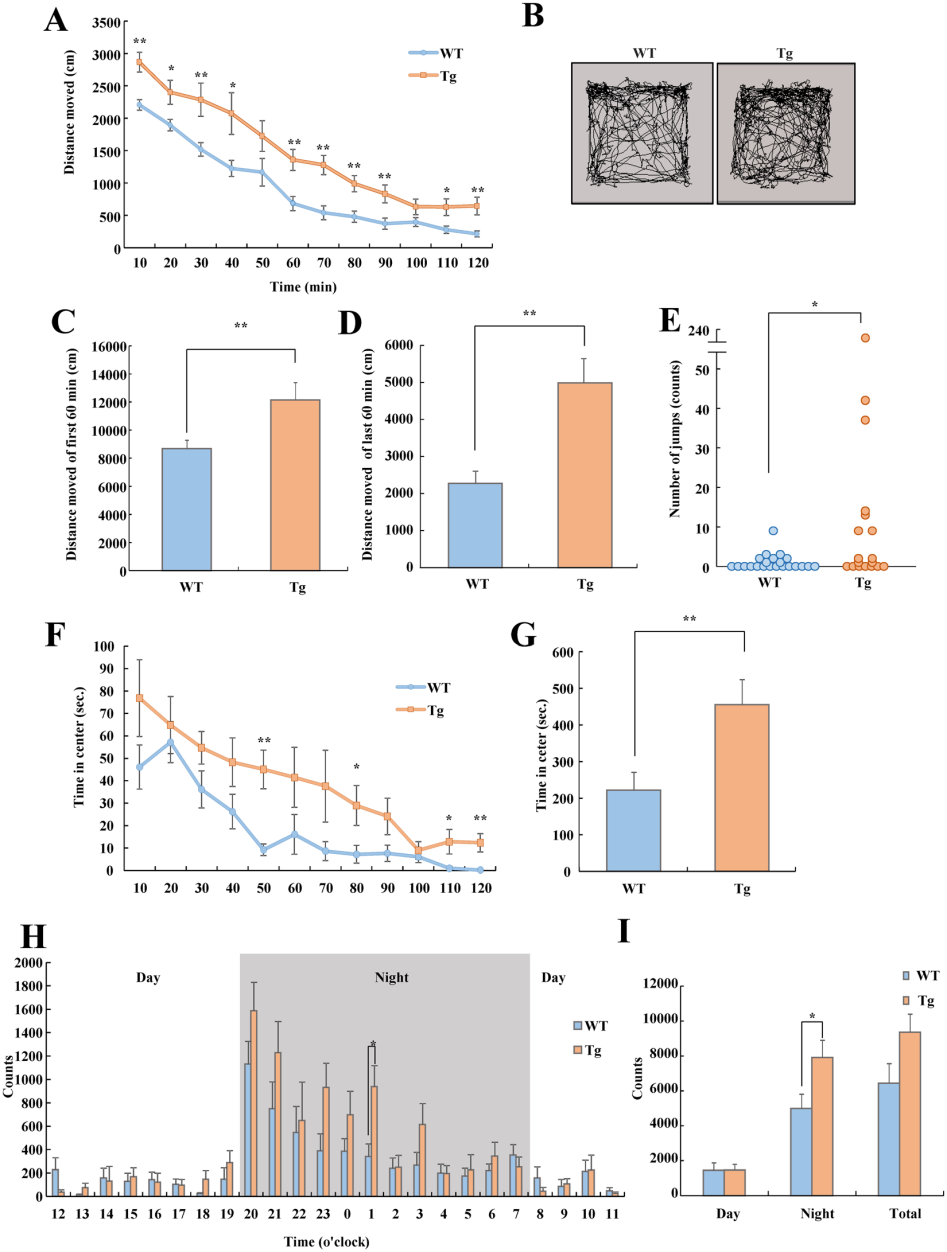
VGF-overexpressing mice exhibited hyperactivity in home cage and novel environment. (**A–G**) Open field test. Mice were placed in an open field, and their locomotion was assessed for 120 min. WT (n = 22), VGF-overexpressing mice (n = 20). (**A**) Total distance was scored for each 10 min period. Genotype; F (1, 40) = 15.524, *p* < 0.001, time; F (11, 440) = 81.235, *p* < 0.001, genotype × time; F (11, 440) = 1.283, *p* = 0.231, two-way ANOVA with repeated measures (0–10: t = −3.894, df = 40, *p* < 0.001, 10–20: t = −2.542, df = 40, *p* = 0.015, 20–30: t = −2.856, df = 40, *p* = 0.007, 30–40: t = −2.555, df = 40, *p* = 0.015, 50–60: t = −3.487, df = 40, *p* = 0.001, 60–70: t = −4.082, df = 40, *p* < 0.001, 70–80: t = −3.386, df = 40, *p* = 0.002, 80–90: t = −2.867, df = 40, *p* = 0.007, 100–110: t = −2.556, df = 40, *p* = 0.014, 110–120: t = −3.104, df = 40, *p* = 0.003). (**B**) Representative images show typical examples of exploring behavior of WT and VGF-overexpressing mice during the first 10 min of the open field test. (**C**) Total distance of first 60 min (t = −3.188, df = 40, *p* = 0.003). (**D**) Total distance during last 60 min (t = −3.807, df = 40, *p* < 0.001). Data are expressed as the mean ± SEM *p < 0.05, **p < 0.01 vs. WT mice (Student’s *t*-test). (**E**) The number of jumps during first 10 min of the open field test. Actual number of jumps observed in individual mice (*p* = 0.020). *p < 0.05 vs. WT mice (Mann–Whitney *U*-test). (**F**) Time spent in the center was scored for each 10 min period. Genotype; F (1, 40) = 8.108, *p* = 0.007, time; F (11, 440) = 14.652, *p* < 0.001, genotype × time; F (11, 440) = 0.937, *p* = 0.504, two-way ANOVA with repeated measures (40–50: t = −4.171, df = 40, *p* < 0.001, 70–80: t = −1.911, df = 40, *p* = 0.027, 100–110: t = −2.219, df = 40, *p* = 0.032, 110–120: t = −3.121, df = 40, *p* = 0.003) (**G**) Time spent in the center throughout 120 min (t = −2.847, df = 40, *p* = 0.007). Data are expressed as the mean ± SEM *p < 0.05, **p < 0.01 vs. WT mice (Student’s *t*-test). (**H,I**) Locomotor activity test. Mice were placed into an open field, and their locomotion was assessed every hour for 1 day after 24 h of acclimatization (n = 8). (H) Locomotor activity throughout the 24-h period. Day: genotype; F (1, 14) = 0.001, *p* = 0.979, time; F (11, 154) = 1.431, *p* = 0.164, genotype × time; F (11, 154) = 0.920, *p* = 0.523, night: genotype; F (1, 14) = 5.112, *p* = 0.040, time; F (11, 154) = 10.352, *p* < 0.001, genotype × time; F (11, 154) = 1.220, *p* = 0.278, total: genotype; F (1, 14) = 3.729, *p* = 0.074, time; F (23, 322) = 14.065, *p* < 0.001, genotype × time; F (23, 322) = 1.569, *p* = 0.049, two-way ANOVA with repeated measures (t = −2.844, df = 14, *p*_1–2_ = 0.013). (**I**) locomotor activity was analyzed separately during the day and night (day: t = −0.026, df = 14, *p* = 0.979, night: t = −2.261, df = 14, *p* = 0.040, t = −1.931, df = 14, *p* = 0.074). Data are expressed as the mean ± SEM, *p < 0.05 vs. WT mice (Student’s *t*-test).

To investigate the effect of VGF overexpression on behavior, we performed the locomotor activity test on mice in a novel environment and home cage. At all time points (every 10 min for 120 min), the level of activity of VGF-overexpressing mice was higher than that of WT mice (Fig. 4A–D). Generally, mice acclimatize in the environment over time. To investigate the spontaneous activity in the novel environment and degree of the habituation of the environment, we investigated the activity during the first and last 60 min. The activity level during both first and last 60 min of the test was higher in VGF-overexpressing mice than WT mice (Fig. 4C,D). Interestingly, a frequent jumping behavior was observed in VGF-overexpressing mice, which was rarely observed in WT mice. During the first 10 min, the number of jumping behaviors in VGF-overexpressing mice (18.60 ± 11.49; n = 20) was higher than that of WT mice (1.04 ± 0.44; n = 22) (Fig. 4E). The time spent in the center is commonly used as the anxiety-related behavior. VGF-overexpressing mice spent more time in the center area in the open field (Fig. 4F,G). In the home cage, the activity of VGF-overexpressing mice did not differ from that of WT mice during the light phase but markedly increased just as the dark phase began (Fig. 4H,I). These results suggest that VGF-overexpressing mice exhibit hyperactivity in both a novel environment and home cage and less anxiety.

### VGF-overexpressing mice exhibit working memory impairment

**Figure 5:**
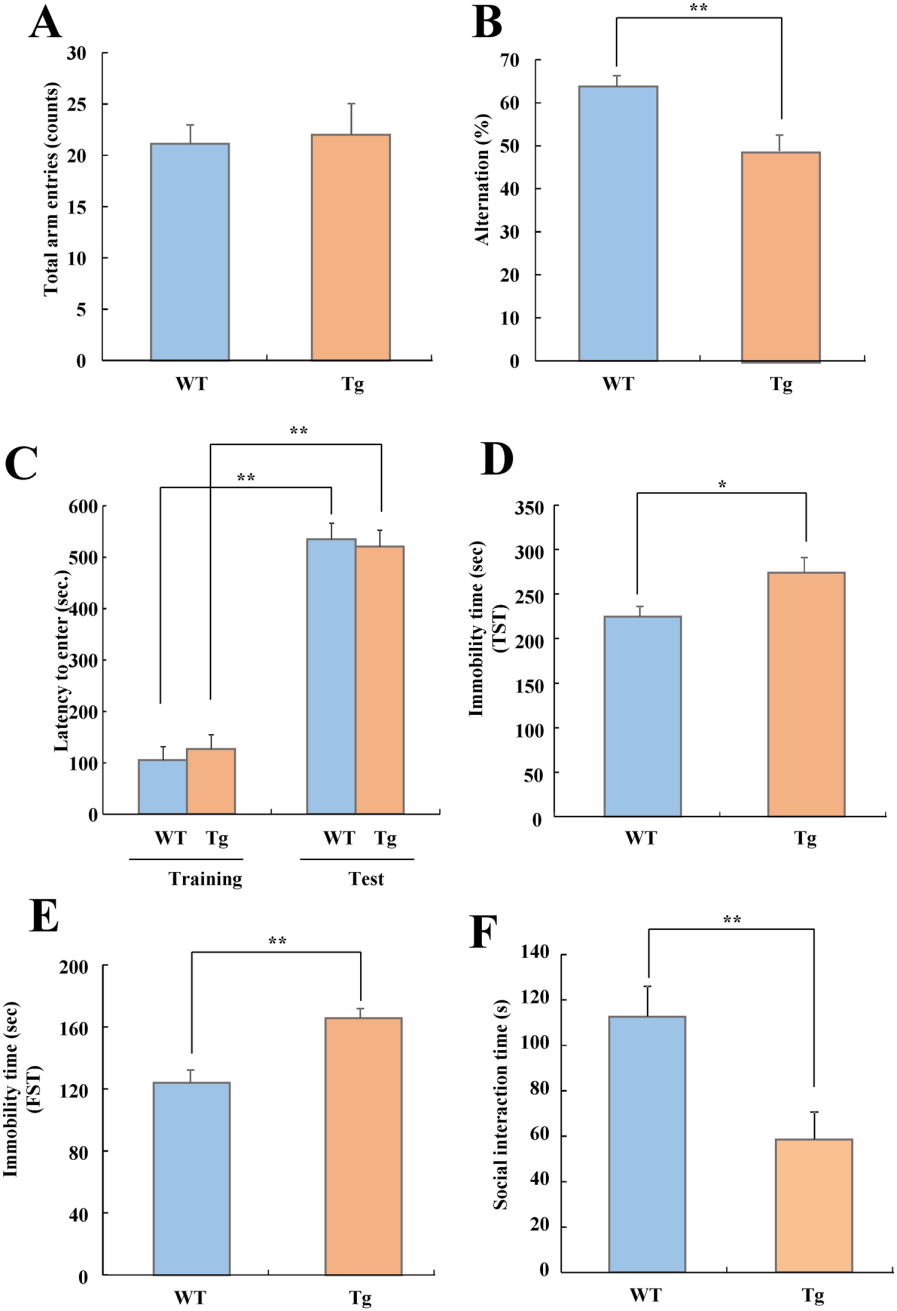
Cognitive function, sociability, and depressive state of WT and VGF-overexpressing mice. (**A** and **B**) Y-maze test. WT (n = 22), VGF-overexpressing mice (n = 19). (**A**) The number of arm entries (t = −0.250, df = 39, *p* = 0.804). Data are expressed as the mean ± SEM, *p < 0.05 vs. WT mice (Student’s *t*-test). (**B**) The percentage of alternation was calculated as (actual alternations/maximum alternations-2) × 100 (t = 3.467, df = 39, *p* = 0.001). (**C**) Passive avoidance test. WT (n = 22), VGF-overexpressing mice (n = 20). Latency to enter the dark compartment in WT and VGF-overexpressing mice at training and test session. Data are expressed as the mean ± SEM. **p < 0.01 vs. training session (paired *t*-test). (**D**) Tail-suspension test of WT and VGF-overexpressing mice. WT (n = 20), VGF-overexpressing mice (n = 19). Immobile time of tail suspension test (t = −2.435, df = 37, *p* = 0.020). Mice were tail suspended with an adhesive tape 50 cm above the floor for 7 min, and immobile time was measured. Data are expressed as the mean ± SEM *p < 0.05 vs. WT mice (Student’s *t*-test). (**E**) Forced swimming test of WT and VGF-overexpressing mice. WT (n = 22), VGF-overexpressing mice (n = 20). Immobile time of forced swimming test (t = −3.974, df = 40, *p* < 0.001). Mice were placed in water for a period of 7 min; only the last 4 min immobility time was measured. Data are expressed as the mean ± SEM **p < 0.01 vs. WT mice (Student’s *t*-test). (**F**) Social interaction test. WT (n = 9), VGF-overexpressing mice (n = 8). Social interaction time measured for 10 min (t = 2.954, df = 15, *p* = 0.010). Data are expressed as the mean ± SEM **p < 0.01 vs. WT mice (Student’s *t*-test).

We evaluated memory functions in VGF-overexpressing mice using the Y-maze test (working memory) and passive avoidance test (fear memory). The number of arm entries did not differ between VGF-overexpressing mice and WT mice (Fig. 5A). However, the alternation of VGF- overexpressing mice was lower than that of WT mice (Fig. 5B). During the training session, VGF-overexpressing mice and WT mice showed a similar latency in crossing to the dark side of the chamber, whereupon they received a single foot shock (Fig. 5C). After 24 h of training, the latency to cross to the dark side increased significantly in both of VGF-overexpressing mice and WT mice compared the training-session response (Fig. 5C). However, there was no difference in latency between VGF-overexpressing mice and WT mice (Fig. 5C). These results indicate that VGF-overexpressing mice exhibit dysfunction in working memory but not fear memory.

### VGF-overexpressing mice exhibit less social behavior and a more depressive state

We evaluated depressive state in VGF-overexpressing mice using the tail suspension test and forced swimming test. For both tests, a higher immobility time was observed in VGF-overexpressing mice (Fig. 5D,E). Using a social interaction paradigm to evaluate the social affiliative behavior of the mice, we observed that the total duration of contact during a 10-min social interaction test in a novel environment was significantly lower in VGF-overexpressing mice than in WT mice (Fig. 5F). This result suggests that VGF-overexpressing mice exhibit fewer social behaviors.

## Discussion

VGF overexpression has been observed in patients with schizophrenia and depression^20^. In this study, we generated VGF-overexpressing mice to investigate the effects of increased VGF expression on brain structure and behavior. VGF-overexpressing mice (line 429) were viable and could be bred normally, unlike homogenous VGF knockout mice^33^. However, three lines (lines 403, 413, and 434) exhibited impaired breeding. VGF may be involved in the survival or reproduction, and viability might vary with degree of the expression level of VGF. We confirmed that VGF-overexpressing mice (line 429) exhibit increased VGF expression in several brain regions by both Western blot analysis and real-time RT-PCR. There was no evident difference in the appearance or body weight between VGF-overexpressing mice and WT mice. However, the brains of VGF-overexpressing mice weighed less than those of WT mice. One report have demonstrated that VGF promotes phosphorylation of Akt and GSK3β^22^. GSK3β is critical for brain development^34^. Thus, the overexpression of VGF may influence brain formation by interacting with these proteins. However, this is the speculation and it is necessary to determine this in the future study.

Morphological abnormalities were observed in the brains of VGF-overexpressing mice, including expansion of the lateral ventricle, a decrease in the size and volume of the whole brain and striatum, and an increase in the neuron density of the striatum, which may be responsible for the reduction of brain weight. However, there were no differences in the morphology of the cerebral cortex or hippocampus between VGF-overexpressing and WT mice. The expansion of the lateral ventricle was greater in the abdominal region, where the striatum is located, than in the caudad region. Although VGF-overexpressing mice exhibited morphological abnormalities in the striatum, the marker of apoptosis (cleaved-PARP) was not detected in the striatum. Because brain atrophy by the apoptosis pathway is accompanied by this marker^35^, these findings indicate that apoptosis was not taking place in the striatum of VGF-overexpressing mice. Clinically, enlarged lateral ventricles and decreased striatal volume have been observed in patients with mental illnesses such as schizophrenia and depression^36–38^. Therefore, VGF may be important for the development and maintenance of striatal morphology. Previously, increased cell density with the decrease in cortical thickness was reported in prefrontal cortex of patients with schizophrenia^39^. It has been guessed that the event was caused by the reduction of cell size^40^. We consider that similar event may occur in striatum of VGF-overexpressing mice. However, we have not investigated the details including axons, dendritic branching or synapse structure. Therefore, we need the further examination to investigate the detailed causes of the striatal tissue abnormality.

To investigate the mechanisms underlying VGF overexpression in the brain, we examined the expression levels of neurotrophic factors known to induce VGF expression^12–14^. However, there were no differences in the expression levels of NGF or BDNF between VGF-overexpressing and WT mice. Although we have not investigated the *Ngf* and *Bdnf* mRNA level and the signaling associated with these factors, these data suggest that the overexpression of VGF may not affect the expression of these factors.

We performed behavioral tests on VGF-overexpressing mice to investigate the role of VGF in behavior. VGF-overexpressing mice exhibited hyperactivity in both a novel environment and a home cage. In addition to hyperactivity, VGF-overexpressing mice spent more time in the center of the open field than WT mice, indicating that VGF-overexpressing mice exhibit less anxiety, although the hyperactivity of VGF-overexpressing mice may affect the score. In Y-maze test, VGF-overexpressing mice showed signs of working memory impairment. Locomotor activity and working memory involve several regions of the brain, including the striatum^41, 42^. Therefore, the striatal abnormality may contribute to the observed locomotor hyperactivity and working memory impairment. In addition, mouse models of mental illnesses such as schizophrenia display hyperactivity and working memory defects^43–46^.

Lengthened immobility time in the tail suspension and forced swimming tests is commonly used as an indicator of depression-like behavior because this behavior is decreased by treatment with antidepressants^27^. Moreover, immobility time is also considered a negative symptom of schizophrenia because this behavior is increased in schizophrenia model mice^47, 48^. Abnormality of the sociality in VGF-overexpressing mice was characterized by a reduction in social behavior compared WT mice in social interaction test, which is the common symptom of model mice of schizophrenia and depression^49–51^.

VGF knockout mice exhibited depressive behavior and memory dysfunction similar to those of VGF-overexpressing mice^15, 23^. Hypomorphic and hypermorphic expression of VGF may produce several common behavioral phenotypes. This is surprising finding, and it is important to discover mechanisms that underlie the common behavioral abnormalities of VGF knockout and VGF-overexpressing mice. In previous reports, hypomorphic and hypermorphic expressions of other factors (e.g. neureglin 1 and BDNF) have produced several common phenotypes^50, 52–54^. The brain region in which BDNF is ablated or overexpressed may be critical to the depressive phenotype. For example, hippocampal BDNF ablation is prodepressant while BDNF ablation in VTA projecting to NAc is antidepressant^55, 56^. The outcomes of VGF-overexpressing mice that are similar to VGF knockout mice could be dependent on the brain region of overexpression. Additionally, it is important to clarify whether the abnormal behaviors of VGF-overexpressing mice are related to schizophrenia or/and depression by evaluating the efficacy of therapeutic drugs for these illnesses. Due to the transgene which contains the CAG promotor, the potential expression of VGF in tissues in which VGF is not normally expressed may be caused. Therefore, it is necessary to consider that the potential expression of VGF in tissues in which VGF is not normally expressed may affect the phenotypes of VGF-overexpressing mice.

We investigated only one line of the VGF-overexpressing mice, thus cannot rule out the effect of transgene integration site on these results, which is the limit of the present study. Therefore, we identified only one integration site, which is located in the 2nd intron of leucine rich repeat and Ig domain containing 2 (lingo 2) gene on mouse chromosome 4, and the expression level of *lingo 2* mRNA was decreased in VGF-overexpressing mice. The mutations of this gene have been associated with body mass, essential tremor, and Parkinson’s disease (PD) in human^57–59^. However, there are no reports on the physiology of this gene. We have not observed the evident tremor in VGF-overexpressing mice. Moreover, previous reports demonstrated that VGF has an anabolic effect from the phenotype of VGF knockout mice and Vgf ^flox/flox^ mice, in which VGF was overexpressed by the stabilization of VGF mRNA^33, 60^. Conversely, VGF-derived peptide TLQP-21 has a catabolic effect^61^. These findings indicate that VGF may have a biphasic effect in the energy balance. Based on these reports, it is possible that the expression balance of several VGF-derived peptides and reduction of lingo2 expression may contribute to the body mass of VGF-overexpressing mice. Additionally, one of the reason why there was the difference between VGF-overexpressing mice and Vgf ^flox/flox^ mice on the phenotype of body mass may be the temporal and spatial VGF expression differences by the difference of promotor. On the other hand, striatal abnormality is implicated with the pathophysiology of Parkinson’s disease in human. However, we consider that a possible lingo2 phenotype may be less consistent with the striatal phenotype of VGF-overexpressing mice, although this possibility could not be ruled out. This reason is that the apoptosis marker (cleaved-PARP) was not detected in the striatum of VGF-overexpressing mice, although neurodegeneration of nigrostriatal dopaminergic neurons occur in patients and animal models of PD^62^. Future investigations are needed to identify and validate specific genes that regulate these abnormalities.

In conclusion, the present study demonstrates that VGF-overexpressing mice exhibit behavioral and morphological abnormalities that may be related to the mental illnesses such as schizophrenia and depression. These findings indicate that VGF may be implicated in the pathogenesis of several mental illnesses and in brain development. This transgenic mouse line may provide a useful model for research on mental illnesses.

## Electronic supplementary material

Supporting information
