# Author Correction: Behavioral abnormalities with disruption of brain structure in mice overexpressing VGF

**DOI:** 10.1038/s41598-018-35362-y

**Published:** 2018-11-20

**Authors:** Takahiro Mizoguchi, Hiroko Minakuchi, Mitsue Ishisaka, Kazuhiro Tsuruma, Masamitsu Shimazawa, Hideaki Hara

**Affiliations:** Molecular Pharmacology, Department of Biofunctional Evaluation, Gifu Pharmaceutical University, Gifu, Japan

Correction to: *Scientific Reports* 10.1038/s41598-017-04132-7, published online 05 July 2017

This Article contains errors in the Materials and Methods section.

Under subheading ‘Animals’,

“The target gene (VGF transgene) was amplified by 35 cycles of PCR using the following primers: VGF forward primer, 5′-CCTACAGCTCCTGGG-3′; VGF reverse primer, 5′-AGAGGGAAAAAGATCRCAGTGGTAT-3′.”

should read:

“The target gene (VGF transgene) was amplified by 35 cycles of PCR using the following primers: VGF forward primer, 5′-CCTACAGCTCCTGGGCAACGTGCTGGTT-3′; VGF reverse primer, 5′-AGAGGGAAAAAGATCTCAGTGGTAT-3′.”

Under subheading ‘Real-time RT-PCR analysis’,

“For VGF, the forward primer was 5′-CAGGCTCGAATCCGAAAG-3′ and the reverse primer was 5′-CTTGGATAAGGGTGTCAAAGTCTCA-3′.”

should read:

“For VGF, the forward primer was 5′-CAGGCTCGAATGTCCGAAAG-3′ and the reverse primer was 5′-CTTGGATAAGGGTGTCAAAGTCTCA-3′.”

and

“For β-actin, the forward primer was 5′-CATCCGTAAAGACCTCTATGCC-3′, and the reverse primer was 5′-ATGGAGCCACCGATCCACA-3′.”

should read:

“For β-actin, the forward primer was 5′-CATCCGTAAAGACCTCTATGCCAAC-3′, and the reverse primer was 5′-ATGGAGCCACCGATCCACA-3′.”

