# Supplementary material for: Behavioral abnormalities with disruption of brain structure in mice overexpressing VGF
Source: Sci Rep. 2017 Jul 5;7:4691. doi: 10.1038/s41598-017-04132-7 (PMC5498671; doi:10.1038/s41598-017-04132-7)
Supplement: Supplementary file 1 — Supporting information [file 41598_2017_4132_MOESM1_ESM.pdf]

Behavioral abnormalities with disruption of brain structure in mice overexpressing VGF

Takahiro Mizoguchi[1], Hiroko Minakuchi[1], Mitsue Ishisaka[1], Kazuhiro Tsuruma[1], Masamitsu Shimazawa[1], Hideaki Hara[1*]

[1]Molecular Pharmacology, Department of Biofunctional Evaluation, Gifu Pharmaceutical University, Gifu, Japan

[*]For reprints and all correspondence: Professor H. Hara, Ph.D., R.Ph., Molecular Pharmacology, Department of Biofunctional Evaluation, Gifu Pharmaceutical University, 1-25-4 Daigaku-nishi, Gifu 501-1196, Japan.

Tel & Fax: +81-58-230-8126

e-mail: hidehara@gifu-pu.ac.jp

Supplemental Materials and Methods

Enzyme-linked immunosorbent assay (ELISA)

Nerve growth factor (NGF) and brain-derived neurotrophic factor (BDNF) levels were measured using standard sandwich ELISA technology by means of the Mouse NGF, BDNF, and EGF ELISA kit (KA0400. KA0331, and KA0544 respectively, Abnova, Taiwan).

Supplementary Fig. 1

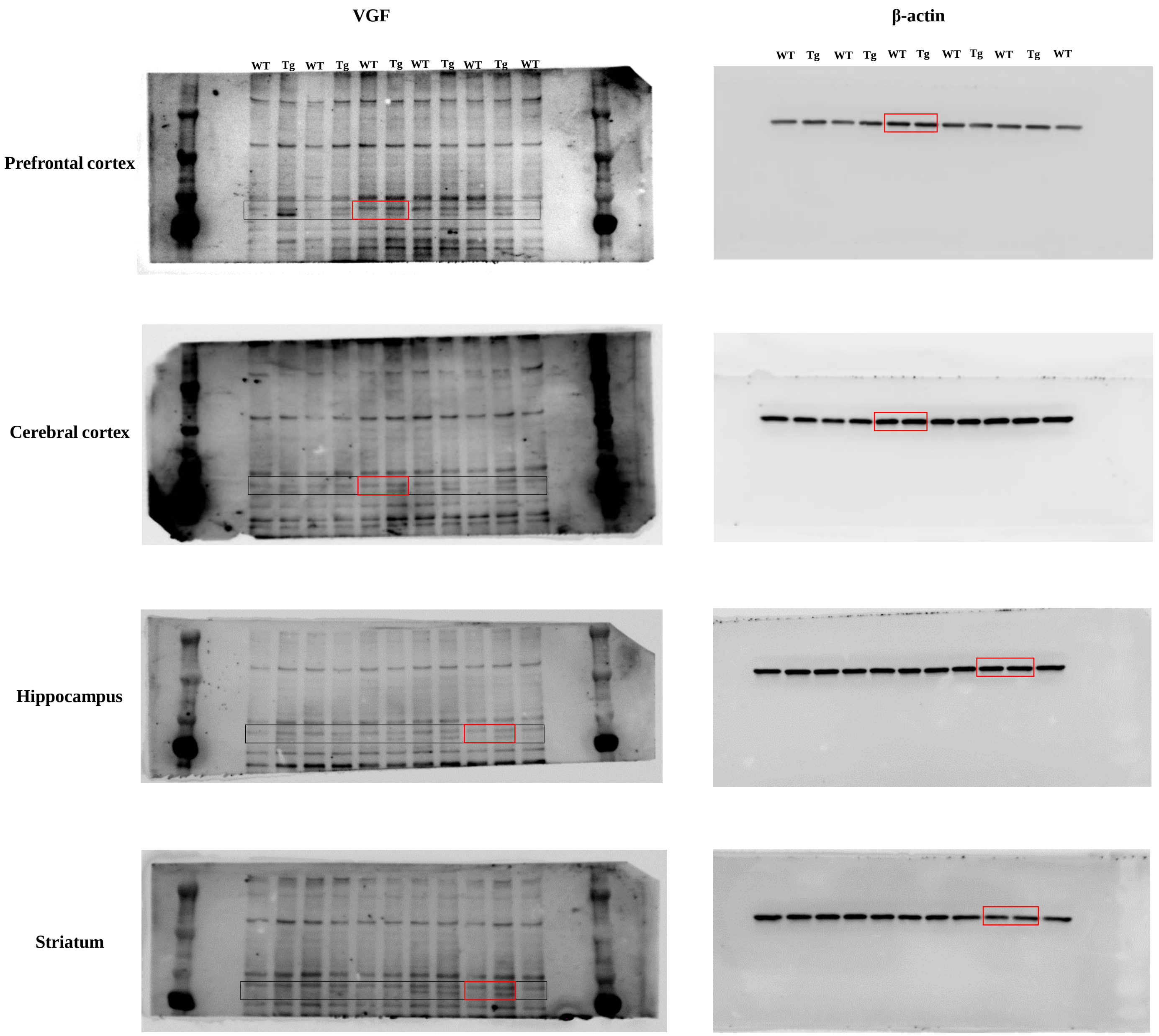

Supplementary Figure 1. Western blot analysis of VGF expression in the several brain regions.

Representative immunoblot showing the VGF expression level in prefrontal cortex, cerebral cortex, hippocampus and striatum. Red squares in the full-length blots are used for the cropped blots in Figure 1.

Supplementary Fig. 2

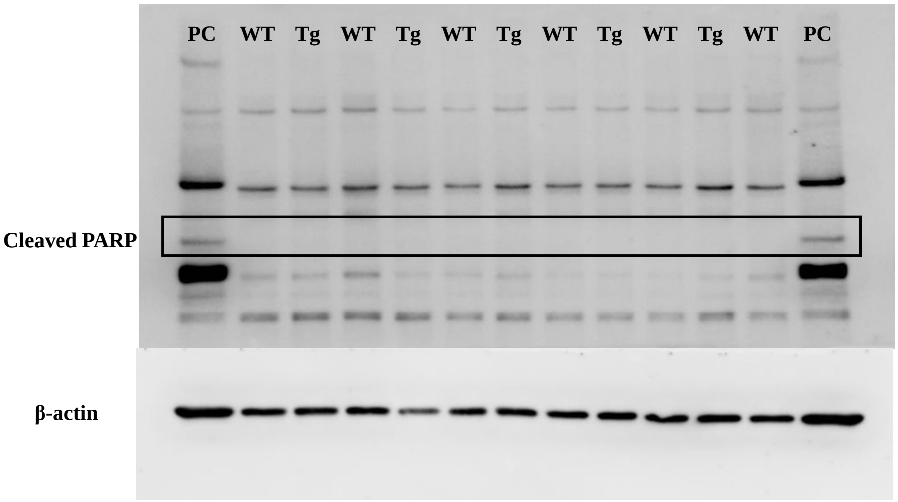

Supplementary Figure 2. Western blot analysis of PARP expression in the striatum.

Representative immunoblot showing the PARP expression level. Western blot did not detect cleaved PARP in WT or VGF-overexpressing mice. NB-1RGB cells treated with UV-A (10 J/cm²) irradiation is the positive control (both ends of lanes, PC).

Supplementary Fig. 3

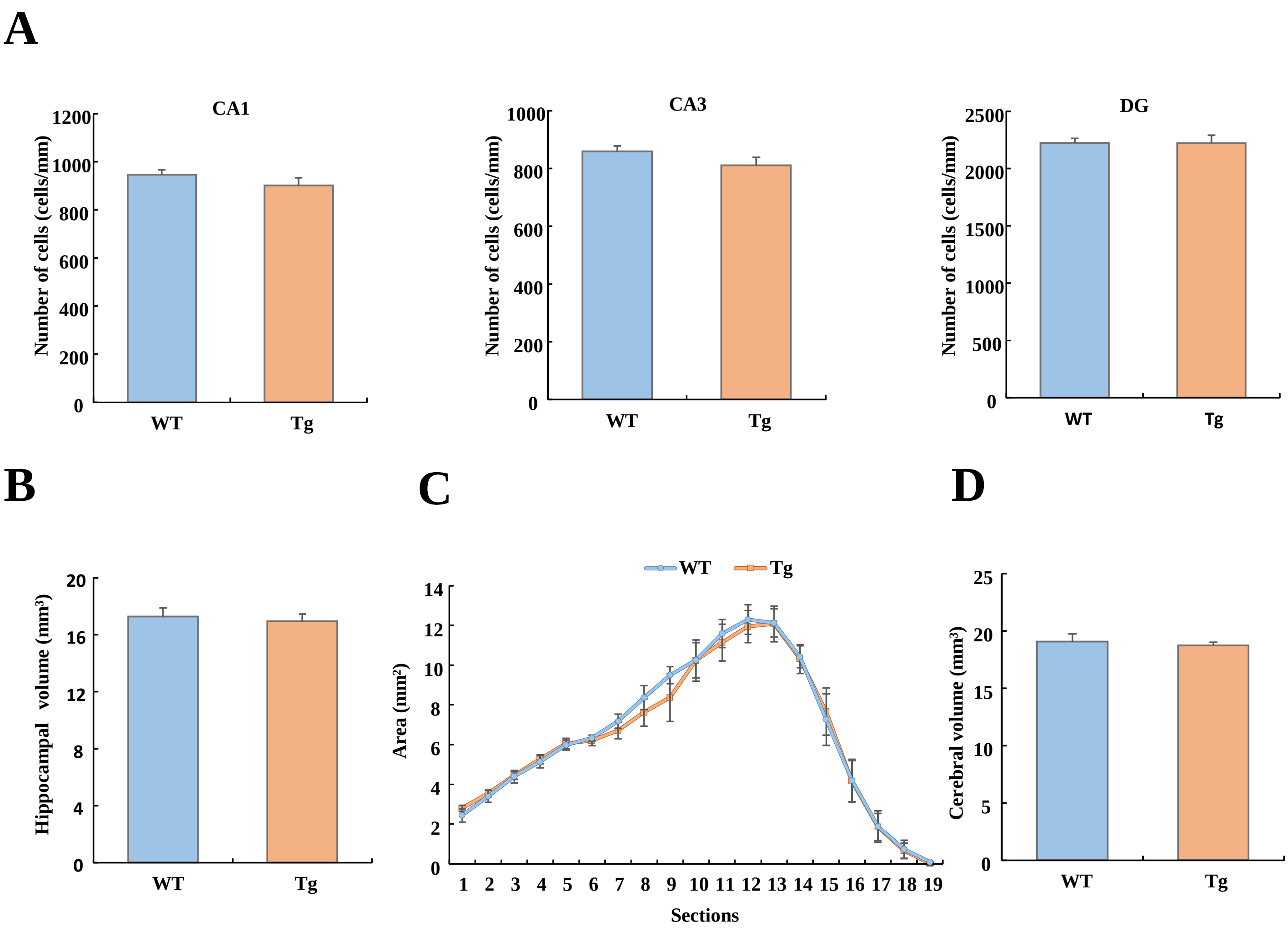

Supplementary Figure 3. Histological analysis of the hippocampus and cortex in WT and VGF-overexpressing mice.

(A-C) Histological analysis of the hippocampus. (A) The number of Nissl-stained cells counted in the CA1, CA3, and DG. Data are expressed as the mean ± SEM (WT, n = 11; Tg, n = 9). (B) Hippocampal volume in WT and VGF-overexpressing mice. (C) The hippocampal region of WT and VGF-overexpressing mice is visualized per section in a rostral to caudal manner. Data are expressed as the mean ± SEM (WT, n = 10; Tg, n = 9). (D) Cortical volume of WT and VGF-overexpressing mice. Data are expressed as the mean ± SEM (WT, n = 5; Tg, n = 6).

Supplementary Fig. 4

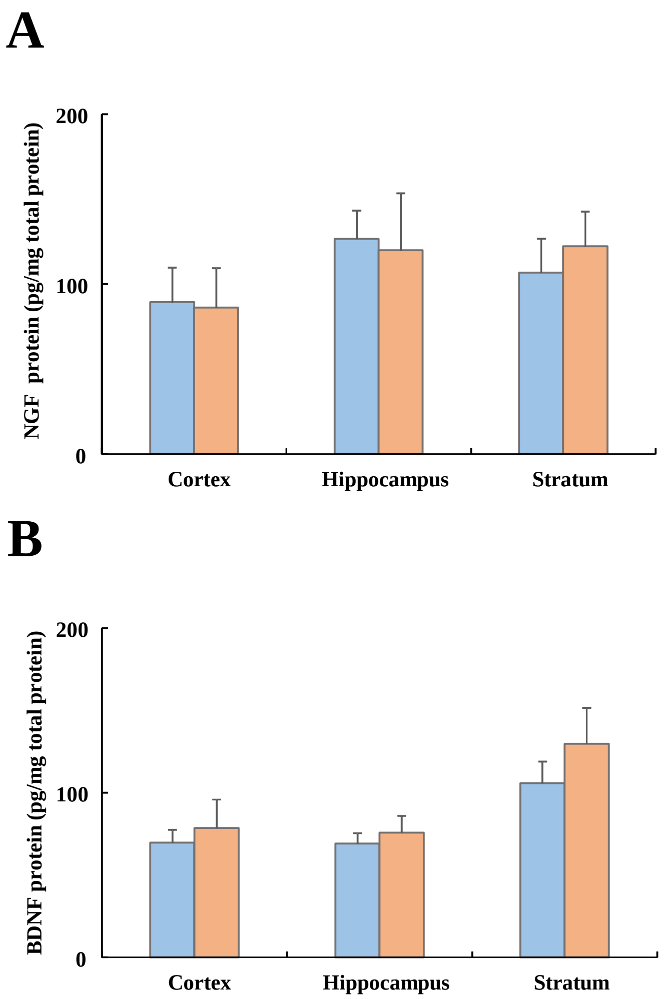

Supplementary Figure 4. Tissue neurotrophic factor levels in brain sections.

Levels of the neurotrophic factors (A) NGF and (B) BDNF were assayed by enzyme immunoassay (EIA). Data are expressed as the mean ± SEM. (WT, n = 4; Tg, n = 6).
